# Facial disability index (FDI): Adaptation to Spanish, reliability and validity

**DOI:** 10.4317/medoral.18054

**Published:** 2012-08-28

**Authors:** Eduardo Gonzalez-Cardero, Pedro Infante-Cossio, Aurelio Cayuela, Manuel Acosta-Feria, Jose-Luis Gutierrez-Perez

**Affiliations:** 1 Resident, Department of Oral and Maxillofacial Surgery, Virgen del Rocio University Hospital, Seville, Spain; 2 Professor, Department of Oral and Maxillofacial Surgery, Virgen del Rocio University Hospital, Seville, Spain; 3 Public Health Technician, South Health District, Seville, Spain; 4 Staff Surgeon, Department of Oral and Maxillofacial Surgery, Santa Lucia University Hospital, Cartagena (Murcia), Spain

## Abstract

Objectives: To adapt to Spanish the facial disability index (FDI) described by VanSwearingen and Brach in 1995 and to assess its reliability and validity in patients with facial nerve paresis after parotidectomy. Study Design: The present study was conducted in two different stages: a) cross-cultural adaptation of the questionnaire and b) cross-sectional study of a control group of 79 Spanish-speaking patients who suffered facial paresis after superficial parotidectomy with facial nerve preservation. The cross-cultural adaptation process comprised the following stages: (I) initial translation, (II) synthesis of the translated document, (III) retro-translation, (IV) review by a board of experts, (V) pilot study of the pre-final draft and (VI) analysis of the pilot study and final draft. Results: The reliability and internal consistency of every one of the rating scales included in the FDI (Cronbach’s alpha coefficient) was 0.83 for the complete scale and 0.77 and 0.82 for the physical and the social well-being subscales. The analysis of the factorial validity of the main components of the adapted FDI yielded similar results to the original questionnaire. Bivariate correlations between FDI and House-Brackmann scale were positive. The variance percentage was calculated for all FDI components. Conclusions: The FDI questionnaire is a specific instrument for assessing facial neuromuscular dysfunction which becomes a useful tool in order to determine quality of life in patients with facial nerve paralysis. Spanish adapted FDI is equivalent to the original questionnaire and shows similar reliability and validity. The proven reproducibi-lity, reliability and validity of this questionnaire make it a useful additional tool for evaluating the impact of facial nerve paralysis in Spanish-speaking patients.

** Key words:**Parotidectomy, facial nerve paralysis, facial disability.

## Introduction

Facial nerve paralysis is the main complication of surgical treatment of parotid gland tumors. As a result, preservation of facial nerve function is a key objective in this type of intervention. Several studies on facial nerve function following parotidectomy show that a temporary paralysis or paresis is common and occurs in between 17 and 64.4% of patients (1-5), with an incidence of permanent paralysis ranging between 0 and 5.5%.

Although the facial nerve is kept intact after surgery, patients experience a facial paresis or transient nerve paralysis which usually has a strong functional and socio-laboral impact in them. Patients frequently complain of impaired speech, difficulty eating, difficulty closing eyelids and/or lips, aesthetic deformity of the face, dribbling, etc. Symptoms improve gradually in days or weeks until complete recovery of facial nerve function is achieved. Quality of life in relation to facial nerve paralysis refers to the subjective assessment patients make of different aspects of their daily life which affect their health condition before they get the full functionality of the facial nerve.

Quality of life is assessed by means of a series of questionnaires or scales which include items or questions distributed into domains or fields and which are analyzed either individually or globally. General questionnaires to measure quality of life like the SF-36 (*Short Form 36 Health Survey*) or the HRLQ (*Health Related Quality of Life*) and even other head and neck-specific surveys have not proved useful to discriminate the true difficulties experienced by patients with facial mobility disorders.

The Facial Disability Index (FDI; in Spanish IDF, *Índice de Discapacidad Facial*) (6) is an instrument widely used in a great number of studies which has not been adapted to Spanish and whose aim is to assess specific quality of life secondary to alterations of facial mimicry. The FDI is a short, self-report questionnaire dealing with psychosocial and physical impairment aspects associated with facial neuromuscular function. There is no other specific instrument in the Spanish language to evaluate patients with facial paralysis after parotid surgery which allows physicians to carry out both the diagnosis of paresis effects and the early and long-term follow up of such impairment. Taking into account this scenario, the aim of our study has been to adapt the original FDI devised by VanSwearingen and Brach in 1995 (6) to our linguistic and cultural milieu, translating it into Spanish and performing the transcultural adaptation in order to provide, not only a specific tool to assess facial paresis after parotid gland surgery but also to evaluate its impact on the quality of life of patients and evaluate the neuromuscular disorders affecting facial mimicry.

## Material and Methods

In a preliminary stage we performed a literature search of the 10 years previous to the onset of our study in order to establish a translation protocol consistent with the latest reports published on this subject. The main researcher contacted via e-mail the crea-tors of the questionnaire and obtained their consent to carry out the study.

-Study stages

The present study was conducted in two different stages: 1) cross-cultural adaptation of the FDI questionnaire and 2) cross-sectional study in a control group to assess its reliability and validity.

The transcultural adaptation was carried out using the translation-retrotranslation technique (7-10). Prior to any step in the proc-ess, the creators of the FDI gave their consent to the development of the present study and were invited to take part in it. The two translators enrolled for the study were two bilingual certified translators whose first or mother tongue is Spanish and have American English as second language. They were responsible for translating the original FDI from American English into Spanish (step 1). Both translators rated the difficulty to find the conceptual equivalents in the translation in a scale from 1 (minimum difficulty) to 10 (maximum difficulty). Then, one of the researchers and an external expert reconciled both translations, that is, they analyzed and compared the differences between them in order to approve a final Spanish draft (step 2). In order to assess the correctness of the translation, the agreed version was in turn retrotranslated into American English (step 3) by a bilingual speaker (American English as mother tongue and Spanish as second language), who did not know about the questionnaire in its original tongue. Translators were asked to make a conceptual and not a literal translation. In a following step, the retrotranslation and the original were compared (step 4) analysing differences and contradictions and an almost definitive Spanish version was written. The comparison criteria were: *different*, when the resulting item lost its original meaning; *literal*, when the result was identical to the original and *similar or conceptual*, when some word changed its meaning but the concept of the questionnaire was maintained. Items were revised and modified according to the researchers’ criteria in order to solve discrepancies. The next step was to perform an analysis of the comprehensibility and equivalence of the final version submitting it to the test of 20 patients with facial paresis after parotidectomy (step 5). After the real application in patients, the last required modifications were made and the definitive adapted FDI was obtained (step 6).

The field survey was conducted from 2008 through 2010 on a series of patients admitted in the Oral and Maxillofacial Surgery Department, Virgen del Rocio University Hospital, Seville (Spain). The Research and Ethics Committee of the hospital gave their consent to perform the study. Inclusion criteria were: incident cases corresponding to diagnosed patients not yet treated for a benign tumor in the superficial lobe of the parotid gland confirmed by means of histological examination by a fine needle aspiration biopsy of the tumoral tissue and an imaging study (CT/MRI), and patients eligible for ablative tumor surgery by means of superficial parotidectomy with preservation of the facial nerve. Exclusion criteria were: patients with a previous history of idiopathic facial paralysis or who suffered from it at the moment of the study, surgical section of one or more branches of the facial nerve during surgery resulting in permanent paralysis, previous history of cerebrovascular accidents, psychiatric or psychomotor disorders which prevent the interview with the patient, illiteracy and regular residence outside the influence area of the hospital (temporary residents); we did not consider the level of education. During the pre-surgery visit, patients were informed of their participation in the study; they received the questionnaires, an acknowledgement letter, written information about the project and the informed consent.

-Questionnaire

**Figure 1:**
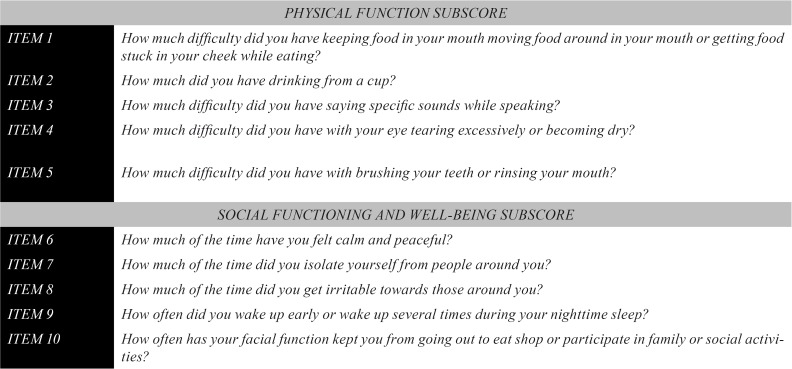
Original version of the FDI.

The FDI (Fig. 1) is internationally validated short-form questionnaire gathering information related to the impact of facial paraly-sis and the physical and social well-being impairment it provokes. It is a specific, short and simple questionnaire easy to be filled in by patients, comprising 10 items or questions distributed into two subscales: physical and social well-being (6). The higher the score obtained in the questionnaire, the better the quality of life of the patient. The aim is to assess disability and the outcome of any intervention in terms of a significant change in the physical disability and social well-being of patients.

-Analysis

Considering that all patients responded to 100% of the items, the feasibility and acceptability of the instrument were taken for granted. We analyzed the following aspects of the translated questionnaire:

The validity of the questionnaire was analysed using the factorial analysis of the main components. The aim was to identify emerging and underlying factors which become evident when we try to group the items or questions answered by patients in the same direction (11). We analysed the correlations found in the answers to the translated questionnaire. This statistical analysis was used to identify the number of dimensions in a group of multivariate items showing the contribution of each item to the specific dimension under study.

Reliability was defined as internal consistency (homogeneity) of the overall questionnaire and of its subscales, calculating Cron-bach’s alpha coefficient of every one of them. Cronbach’s alpha coefficient is the adjusted mean of the correlations between items or questions included in the scale. The expected alpha coefficient was estimated at 0.70; so it was considered that alpha values above than this value were sufficient to ensure the reliability of the scale and to consider that the instrument would calculate consistent and stable measurements.

In order to test the construct validity of the translated questionnaire, we assessed the relationship between the results of the translated questionnaire which evaluated patients’ disability and their true physical impairment using the international House-Brackmann scale (H-B) (12). This scale is a widely-validated standard method (13) to measure facial nerve function, control its evolution over time, and assess recovery and effects after treatment. H-B scale classifies the degree of paresis into 6 levels, from I (no paresis) to VI (total paresis). The total score is obtained by adding the result of each of the 5 branches in the facial nerve, thus obtaining an interval of values which may range from 6 to 36. Pearson’s correlation coefficient was used to determine such bivariate correlation. The result of each of the subscales obtained in the factorial analysis of the main components was compared with the physical examination.

Finally, we determined whether the specific translated questionnaire proved more valid than other general instruments measuring quality of life which have already been translated into Spanish and are widely validated and employed. In order to do so, we compared the FDI and the SF-36 survey (14) once we had asked the © 2011 QualityMetric for the required permission. The SF-36 survey is a standardized self-report instrument including 8 dimensions. We used the physical dimension to compare it with the H-B scale. Pearson’s correlation coefficient was used for such purpose.

Our hypothesis was to establish the lack of correlation or a slight correlation between the SF-36 survey and the H-B scale, comparing it with the correlation between the FDI, the physical subscale of the SF-36 survey and the H-B scale. A value of p<0.05 was considered statistically significant. Data were analysed using the software package SPSS v.15.0 for Windows.

## Results

From January 2008 through December 2010 we carried out a prospective study of the data corresponding to 79 patients who had undergone conservative superficial parotidectomy with preservation of the facial nerve for pleomorphic adenoma of the parotid gland superficial lobe and met the inclusion criteria. Mean age of patients was 40 yrs in an interval between 24 and 81 yrs. Patients were handed the FDI questionnaire three months after the intervention.

**Figure 2:**
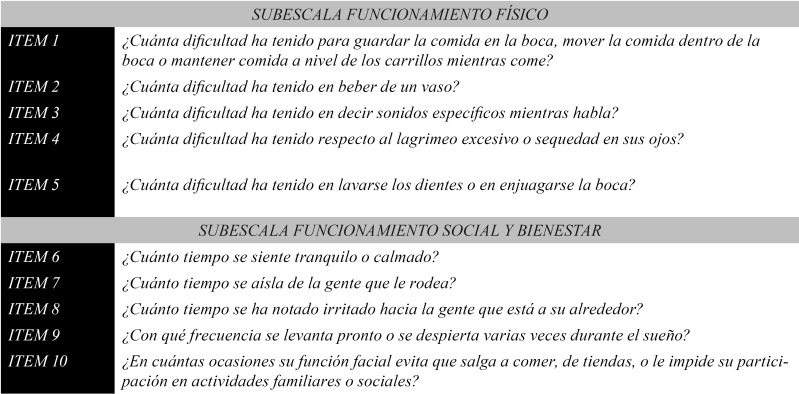
Spanish adapted version of the FDI.

The difficulty of the translation was rated by 2 translators with a mean score of 3. In the end, once the definitive adaptation was evaluated we obtained the complete equivalence in all the items in the questionnaire. Figure 2 shows the final translation of each of the questions in the FDI. For example, some changes were introduced in item 1, “*How much difficulty did you have keeping food in your mouth, moving food around in your mouth or getting food stuck in your cheek while eating?*”, which was translated into Spanish as “*¿Con qué dificultad ha mantenido la comida en la boca, ha movido la comida en el interior de tu boca o ha apartado la comida a un lado de la boca mientras comías?*”. Nevertheless, the final adaptation was: “*¿Cuánta dificultad ha tenido para guardar la comida en la boca, mover la comida dentro de la boca o mantener comida a nivel de los carrillos mientras come?*”. Likewise, item 4: “*How much difficulty did you have with your eye tearing excessively or becoming dry?*”, was translated as “*¿Con qué dificultad sus ojos han lagrimeado excesivamente o se han secado?*”, but the final adaptation: “*¿Cuánta dificultad ha tenido respecto al lagrimeo excesivo o sequedad en sus ojos?*”, was less confusing for patients and explained more clearly the physical disorder it attempted to measure (eye hydration in patients with paresis of the ophthalmic branch of the facial nerve).

Reliability was measured using Cronbach’s alpha coefficient both for the complete scale and for the two dimensions (physical and social well-being functions) established by the analysis of the main components. The data obtained showed adequate reliability at 3 months after surgery. Cronbach’s alpha coefficient was 0.83 for the complete scale and 0.77 and 0.82 for the physical and social well-being subscales, respectively. In an attempt to improve homogeneity, we calculated Cronbach’s coefficient excluding in each case one of the items. For all and every one of the items we obtained lower reliability levels than for the global scale ( Table 1).

**Table 1:**
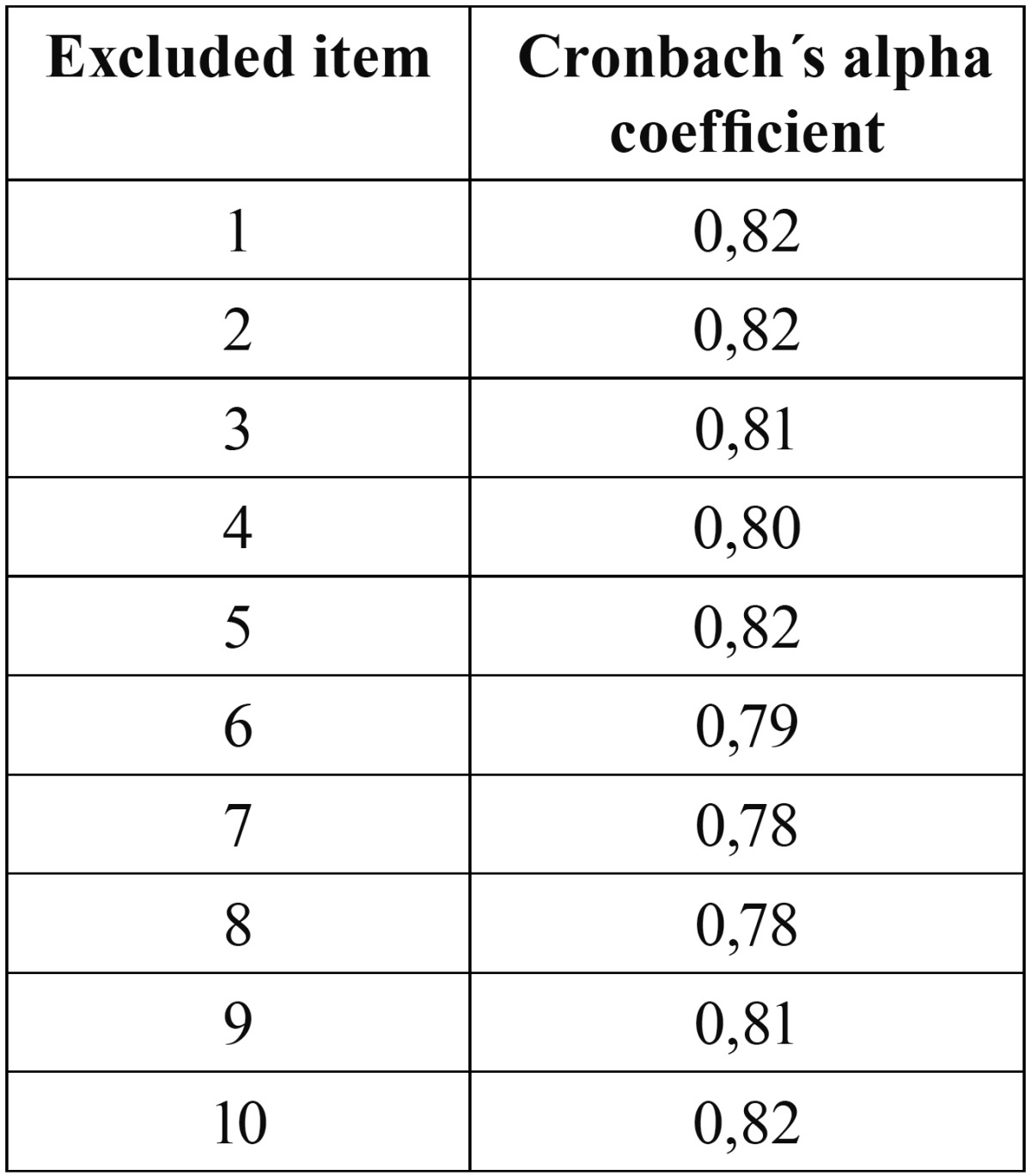
Assessment of reliability and internal consistency (Cronbach’s alpha coefficient) of the adapted FDI, excluding each one of the items.

As regards the analysis of the main components, we carried out a factorial analysis with Varimax rotation and factor extraction with an eigenvalue >1; that is, the analysis of the components should show more variance in the overall instrument than any of the items in the questionnaire. This analysis confirmed that the items included in the physical subscale formed a homogeneous group, clearly apart from the social well-being subscale.  Table 2 highlights those values >0.5 showing the separation between the first component (physical subscale) and the second one (social well-being subscale).

**Table 2:**
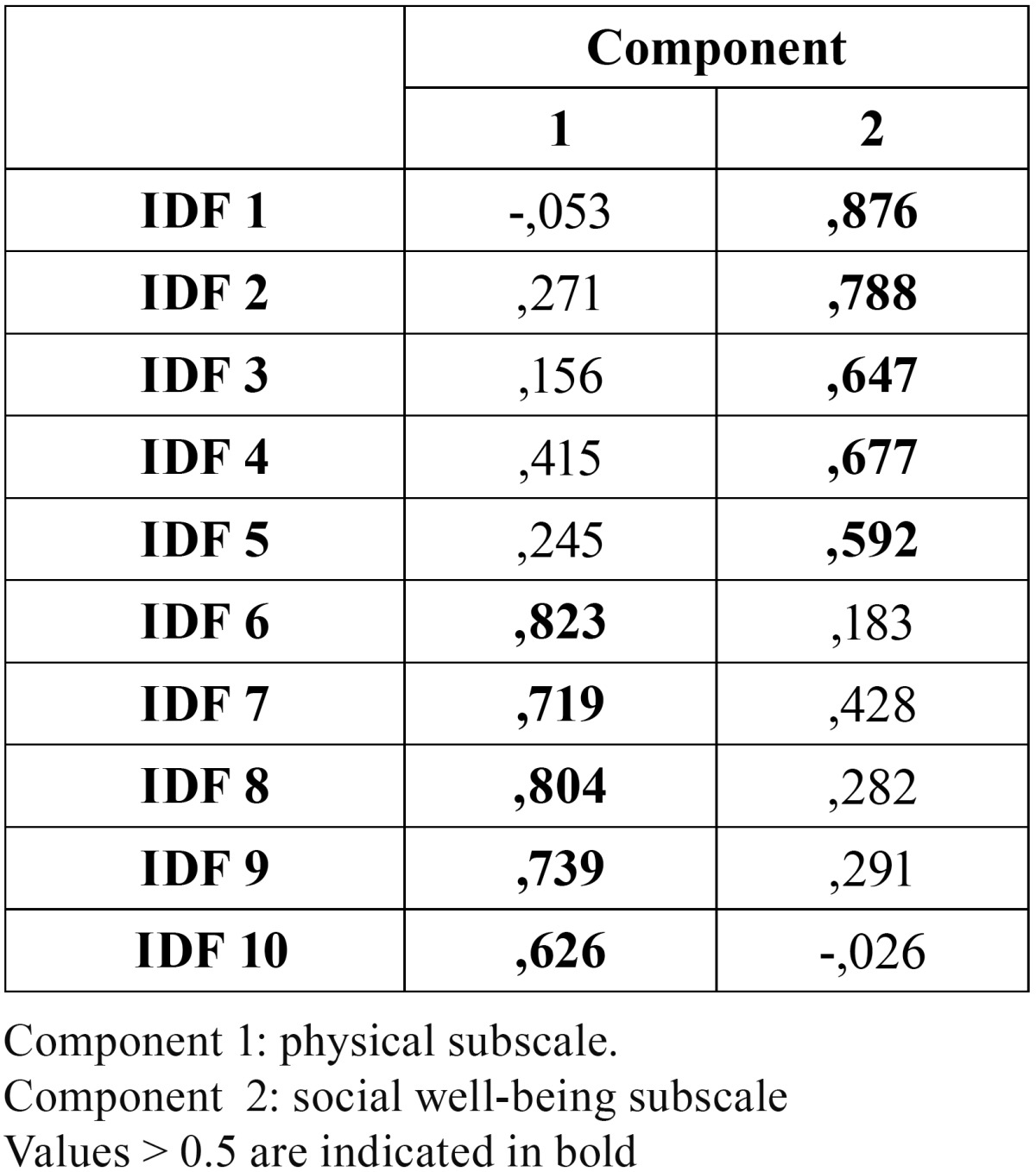
Analysis of the main components of the FDI using rotated component matrix (factor extraction method with analysis of main components and Kaiser Varimax rotation).

The SF-36 survey was filled in by 34 patients.  Table 3 shows bivariate correlations between FDI and H-B scale, and between SF-36 survey (physical function dimension) and H-B scale. As we expected, when we compared the results of the FDI with the true physical dysfunction of the patient measured by means of the H-B scale, we observed a statistically significant correlation between the physical subscale and the clinical diagnosis of facial paresis measured by means of the H-B scale ( Table 3, item a). The analysis of the correlation between the total FDI and the H-B scale revealed a lower correlation than the one observed with the physical subscale on its own ( Table 3, item b). As we hypothesized, a direct relation was not observed between the physical scale in the SF-36 survey and the true physical function of the patient measured by means of the H-B scale ( Table 3, item c), the way it is observed in the case of FDI (physical subscale).

**Table 3:**
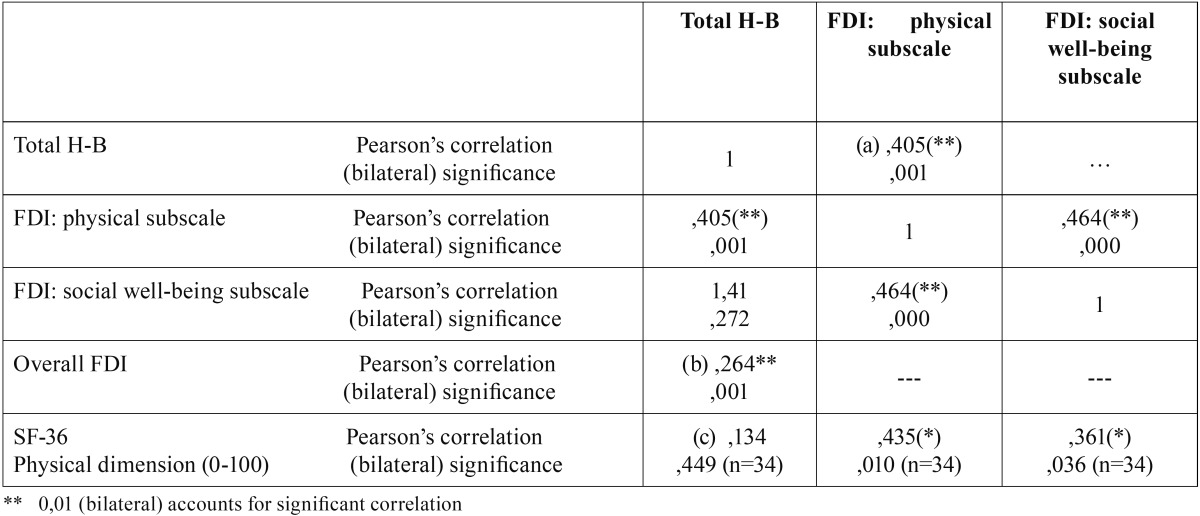
Bivariate correlations between the FDI, H-B scale and SF-36 survey.

 Table 4 shows the analysis of variance percentage revealed by each of the FDI components, which coincides with the validation of the original instrument. The variance percentages of the first and second components were 31.12 and 29.96, respectively.

**Table 4:**
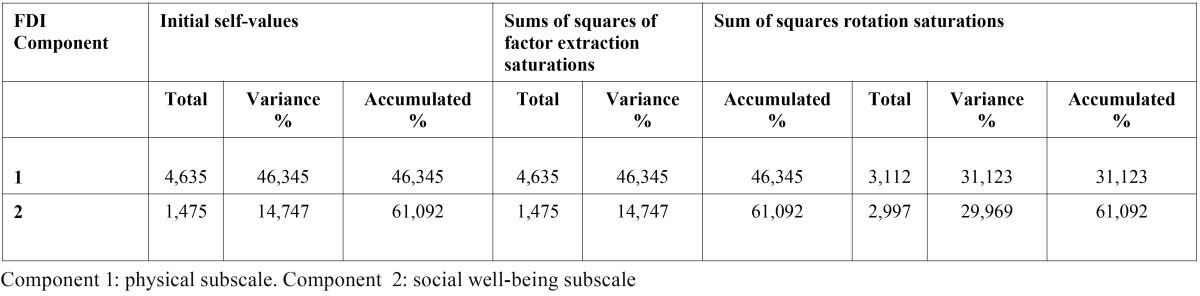
Variance percentage expressed for each one of the FDI components.

## Discussion

The results of the present study suggest that the Spanish adapted FDI is equivalent to the original questionnaire and shows similar reliability and validity as well as similar limitations. Cronbach’s alpha coefficients of the scales and dimensions of the adapted FDI are similar to those of the original questionnaire with values ranging from 0.70 to 0.90. In order to boost the research we have conducted a prospective study of homogeneous incident cases, all of them with the same type of tumor in the same anatomical location and treated with the same surgical technique.

As regards reliability, the FDI assessed quality of life accurately and with adequate internal validity. Reliability surpasses 0.7 (0.83), a level considered adequate to carry out comparisons and to monitorize facial disability in the same patient with self-report instruments and shows a degree of internal consistency similar to that obtained by means of other questionnaires which comprise the same number of items (15). This confirms that the internal validity of the questionnaire has not been altered by the translation process. Unlike the original instrument (6), we observe a slightly lower reliability in the physical dimension (0.77) than in the social well-being dimension (0.82), although in view of the small difference it is not considered relevant. In any case, both values are valid to state the reliability of the FDI.

To carry out factorial analysis, we have used a different sample than to perform the validation of the original instrument, as far as size, sampling technique, language and culture are concerned. Both samples are heterogeneous and comprise different types of people and the sampling technique has been non probabilistic, as it is not a requirement for validation. Therefore, as the results obtained are similar to those obtained in the analysis of the original questionnaire, we can affirm that the questionnaires measure the same, despite the differences between the samples. Similarity is observed in both factor groups, almost identical, which confirms the hypothesis that the cross-cultural adaptation does not alter the questionnaire. After calculating Cronbach’s alpha coefficient discarding 1 item in each case, we found that the global scale of the FDI was even more reliable for the overall instrument than for each isolated item.

Taking into account the lack of a standardized measurement of facial paresis, we used H-B scale to validate the construct (12). Such validity has been proved for the correlation between the FDI and the clinical measurement of facial movement ( Table 3). This correlation also confirmed the conceptual relationship between dysfunction and disability, which underlies facial function recovery. The expected correlation was higher between the physical subscale and the H-B scale (0.405) ( Table 3, item a) in comparison with a global measurement of the FDI and the H-B scale (0.264) ( Table 3, item b), which supports the validity in the sub-scales format.

However, agreeing with our hypothesis, unlike the case with the translated questionnaire, no correlation was observed between the physical dimension of the SF-36 survey and the clinical measurement of facial paresis. This may be explained by the lack of specificity of this latter survey to evaluate facial neuromuscular dysfunction ( Table 3, item c). The SF-36 survey has not proved useful in the evaluation and follow-up of patients as it measures different aspects of physical disability and focuses on other health problems apart from facial neuromuscular dysfunction.

The Spanish translated and adapted FDI operates as a specific questionnaire providing valuable information to evaluate facial neuromuscular dysfunction. The objective assessment of quality of life in relation to facial function using validated instruments may play a key role in the diagnosis and follow up of our patients (16). In this sense, the FDI can be firstly used as a diagnostic instrument and in a subsequent stage as a follow-up tool after treatment or surgery (6). The FDI is a short, simple and easy self-report instrument which can be administered in about 4 minutes. It has been widely accepted by patients, as the translation is comprehensible and is adapted to Spanish language. Researchers have never been required to explain the items or questions during the completion of the questionnaire and physicians have never complained about incomprehensible expressions or difficulty in its use.

Due to the simplicity of most of the items in the questionnaire, a literal translation was preferred for half of them (items number 2,3,5,7 and 8) and a conceptual one for the other half (items number 1,4,6,9 and 10). The translations made separately by the bilingual translators showed almost no differences when compared. The descriptive, clear and concise language of the original questionnaire favored the almost complete agreement between translators. Under no circumstance has the meaning of the questions been modified in order to maintain the object of the question in the original instrument. Neither has it been considered appropriate to create any new item or modify the meaning of any of them. During the pilot study and during the validation process, we have not found any problem with the questions; none of them were misleading or difficult to understand. None of the patients has required any further explanation to fill in the overall questionnaire.

To sum up, the results of the present study show that the Spanish adapted FDI is reliable and valid instrument both for research and for application in daily clinical practice. It is a useful tool to assess the impact of facial disability associated to facial paralysis /paresis following parotidectomy; it is accessible to Spanish speaking patients and physicians involved in the treatment and follow-up of these patients. Future work in this field should focus on the application of this instrument to analyze the different facial neuromuscular disorders and to optimize the management, treatment and rehabilitation of facial paresis or paralysis in the long term.
